# Oxidative Stress Is Differentially Present in Multiple Sclerosis Courses, Early Evident, and Unrelated to Treatment

**DOI:** 10.1155/2014/961863

**Published:** 2014-03-26

**Authors:** Maira Gironi, Bruno Borgiani, Enrica Mariani, Cristina Cursano, Laura Mendozzi, Rossella Cavarretta, Marina Saresella, Mario Clerici, Giancarlo Comi, Marco Rovaris, Roberto Furlan

**Affiliations:** ^1^INSPE, Ospedale San Raffaele, Via Olgettina 60, 20132 Milano, Italy; ^2^CAM, Centro Polidiagnostico, Viale Elvezia Angolo Via Martiri delle Foibe 1, Monza, Italy; ^3^Fondazione IRCCS, S. Maria Nascente, Don Gnocchi, Via Alfonso Capecelatro 66, 20148 Milano, Italy

## Abstract

*Background*. Oxidative stress is well documented in multiple sclerosis (MS) lesions, but its correspondence at peripheral level is still controversial. * Objective*. To evaluate peripheral oxidative stress markers in MS patients. * Methods*. We studied total blood levels of Coenzyme Q_10_ (CoQ_10_), oxidized and reduced forms of glutathione, malondialdehyde, reactive oxygen species (ROS), anti-oxidized-low-density lipoproteins (anti-oxLDL) antibodies, and antioxidant power (PAO) in 87 patients with different MS clinical phenotypes and in 77 controls. * Results*. CoQ_10_ was lower whereas anti-oxLDL antibodies titer was higher in MS patients than in controls. The benign variant of MS displayed both higher CoQ_10_ and higher anti-oxLDL than other MS clinical variants. Female patients had lower CoQ_10_ and PAO and higher ROS than male patients. Differences were greater in younger patients with shorter disease duration. Surprisingly, there was no difference for these markers between treated and untreated patients. * Conclusion*. We found lower antioxidant agents and higher anti-oxLDL antibodies in MS, and the highest antibody titers occurred in the benign form. We suggest that natural anti-oxLDL antibodies can be protective against MS, saving blood brain barrier integrity. Our findings also suggest that milder MS is associated with a distinct oxidative stress pattern, which may provide a useful biomarker of disease prognosis.

## 1. Introduction

Neurodegeneration in multiple sclerosis (MS) is a multifactorial process manifesting from the very onset of the disease [1, 2]. While, in the early stages of MS, neurodegeneration is mainly driven by inflammation [3], later in the course of the disease several interacting factors are involved. Among well-known and less well-documented players, mitochondrial dysfunction seems to have a crucial role [4, 5]. Mitochondrial changes in MS include altered distribution and structure, together with biochemical and molecular abnormalities [4, 6–9]. Mitochondrial damage is caused by several factors, including oxidative stress [10–14]. Oxidative stress can arise in a biological environment whenever there is an imbalance between reactive oxygen species (ROS) production and the cell's buffering capacity; this imbalance results in oxidation of proteins, lipids, and DNA [9, 15]. ROS are natural bioproducts of oxidative phosphorylation [9, 16] but can also be generated by activated inflammatory cells, including macrophages and microglia [17–19]. Just as activated macrophages and microglia are an important source of ROS, oxidative stress can, in turn, activate key factors (such as nuclear factor k Beta) that upregulate proinflammatory gene expression [20]. Thus, an autotoxic loop is sustained [21]. Accordingly, studies of oxidative stress in MS have dramatically increased in recent years [10, 22, 23]. Even though evidence of oxidative stress damage in MS appears unequivocal, the assessment of oxidative stress biomarkers has yielded inconsistent results. The concentration of glutathione, a major antioxidant agent [10], is increased according to some authors but decreased according to others [24]. Similarly, uric acid, a powerful nonenzymatic antioxidant, was reported normal by Kastenbauer et al. [25], decreased by Miller and colleagues [26], and increased by Amorini et al. [27].

These discrepancies may be due to a number of factors: first, the use of different samples (CSF, plasma, and peripheral blood cells) and the application of different laboratory techniques; second, the choice of selected groups of oxidant or antioxidant markers is likely to offer a limited and biased view rather than a general overview of the oxidative stress phenomenon. Because of the high redundancy of the antioxidant system and the dual role played by some antioxidant scavengers, the decrease of one marker could be secondary and possibly a compensatory phenomenon, rather than primary. Finally, some inconsistencies could be due to the different clinical phenotypes of the patients investigated.

Different clinical phenotypes of MS are characterized by distinct histopathological features and, we hypothized, also by distinctive oxidative stress patterns [28]. To investigate this hypothesis while avoiding, as much as possible, the aforementioned confounding factors, we have performed a pilot trial study comparing the levels of several oxidant and antioxidant biomarkers in a large sample of patients with different clinical variants of MS and in healthy control subjects.

Furthermore, we assessed the same markers in patients exposed or nonexposed to immunosuppressive treatment. Consistent with a previous study of oxidative stress molecules in other neurodegenerative diseases [29], we chose a panel composed of the following markers: Coenzyme-Q10 (CoQ_10_); total (GSTot), oxidized (GSSG), and reduced (GSH) forms of glutathione; malondialdehyde (MDA); reactive-oxygen-species (ROS); anti-oxidized-low-density lipoproteins antibodies (anti-oxLDL); and antioxidant power (PAO).

## 2. Subjects and Methods

Eighty-seven patients affected by well-established relapsing-remitting (RR, *n* = 32), benign (BB, *n* = 13), primary (PP, *n* = 20), or secondary progressive (SP, *n* = 22) MS [30] were recruited for the present study (MS Center Fondazione Don Carlo Gnocchi, Milan, Italy, and CAM Polidiagnostic Center, Monza, Italy) between July 2011 and February 2013. Patients with relapsing-remitting course and an Expanded Disability Status Scale (EDSS) score ≤ 3.0 after 15 years of disease were classified as* Benign* MS patients [31]. All MS patients had to be free of relapse or disease progression in the past 30 days. We excluded patients who were treated with mitoxantrone, cyclophosphamide, or steroids, or supplemented with nutraceutical drugs or vitamins during the 3 months before blood drawing. We also excluded patients with clinically or radiologically isolated syndromes. At the time of the study, 20 patients were being treated with beta-interferons, 7 with glatiramer acetate, and 7 were under other treatments (4 on natalizumab, 2 on azathioprine, and 1 on low dose naltrexone). Subjects with serious or unstable medical conditions, including cardiovascular, pulmonary, hepatic, gastrointestinal, renal, and metabolic diseases, malignancies, or diabetes, were excluded from the study. After obtaining informed consent, blood samples were collected in the morning after breakfast and immediately delivered to the central laboratory. Complete neurological examination with EDSS rating was performed in all subjects. The demographic data of all subjects are reported in Table 1. There were no significant age differences among MS patients subgroups (Table 2) or between HC subjects and MS patients (Table 1). The gender distribution was analyzed using a chi-square test and there was no significant difference between MS patients and HC (*P* = 0.5). Table 2 shows a different gender and (as expected) drug distribution across MS subgroups.

We also studied seventy-seven healthy age- and sex-matched controls (HC).

### 2.1. Determination of Oxidative Stress Parameters

Whole blood was collected in vacutainer tubes containing ethylenediaminetetraacetic acid (EDTA) (Becton Dickinson & Co., Rutherford, NJ, USA). Blood sample was centrifuged at 2500 rpm for 5 minutes to obtain serum for the detection of CoQ_10_, MDA, and anti-oxLDL. Plasma was used to measure PAO and ROS. An aliquot of whole blood was used for detection of GSTot, GSSG, and GSH.

#### 2.1.1. Coenzyme Q_10_

CoQ_10_ was determined by isocratic HPLC and UV detection. CoQ_10_ is released by protein precipitation and concentrated by solid phase extraction.

#### 2.1.2. Malondialdehyde

Malondialdehyde (MDA) was determined by isocratic HPLC and fluorescence detection. Sample preparation is based on a protein precipitation step, followed by derivatisation. The resulting fluorophore is specific and detectable at very low levels.

#### 2.1.3. Glutathione

Glutathione (GSTot), in its reduced (GSH) and oxidized (GSSG) form, was measured by HPLC with fluorescence detection. Sample preparation is based on protein precipitation and derivatisation. After precipitation, the sample is split into two portions. One aliquot is derivatised immediately for the determination of GSH; the second aliquot is reduced chemically before derivatisation, which leads to the detection of both oxidized and reduced glutathione. Inclusion of an internal standard minimizes any analytical variation.

#### 2.1.4. Reactive Oxygen Species

We assessed reactive oxygen species by d-ROMs test (Diacron). ROMs (primarily hydroperoxides and ROOH), in the presence of iron (which is released from plasma proteins by an acidic buffer kit), generate alkoxyl (R-O^•^) and peroxyl (R-OO^•^) radicals through the Fenton's reaction. Such radicals, in turn, oxidized an alkyl-substituted aromatic amine which acquires a *t* photometrically detectable pink color.

#### 2.1.5. Antioxidant Power

Serum antioxidant levels were measured using the Total Antioxidant Power Kit (Oxford Biomedical Research, Oxford, MI, USA). The evaluation of serum antioxidant levels is based on the reduction of Cu++ into Cu+. The reduced form of copper gives rise to a stable complex with a chromogenic reagent and shows maximum absorbance at 450 nm. Known concentrations of uric acid are used to create a calibration curve. The values are expressed as *μ*M copper reducing equivalents (CRE).

To assess anti-oxidized low density lipoproteins antibodies, we used IMTEC-oxLDL-Antibodies Ig (GM) (IMTEC-Human Wiesbaden, Germany). The test is based on simultaneous incubation of serum samples with oxLDL immobilized into microtiter wells and native LDL immobilized on pins of the microplate cover.

The binding of antibodies anti-oxidized LDL (anti-oxLDL) from patient serum is detected by an anti-human HRP conjugate and the subsequent reaction of a chromogenic substrate.

### 2.2. Statistical Analysis

The normal distribution of all measured data was ascertained using the Kolmogorov-Smirnov test. Each oxidative stress biomarker was normally distributed (all *P* > 0.05). To assess group differences in oxidative stress biomarkers between MS and HC, a Student's *t*-test for unpaired observations was used. Group differences between different MS courses and HC concerning oxidative stress biomarkers were assessed with a one-way ANOVA, followed by Tukey HSD or Dunnett *t* post hoc analyses. The relationships between 2 continuous variables were examined by Pearson's correlation (*r*). All statistical analyses were performed with the SPSS statistical software package version 15.0 (SPSS, Chicago, IL, USA). Statistical significance was taken to be at the two-tailed 0.05 level.

## 3. Results

The levels of oxidative stress biomarkers in the studied population are shown in Table 3.

**Figure 1:**
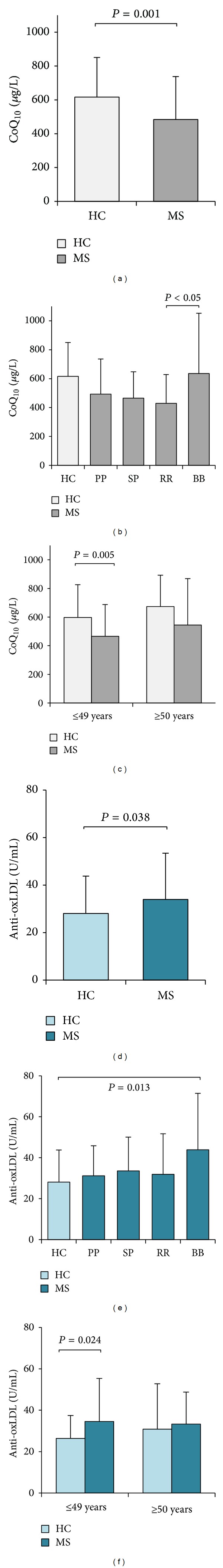
Bar plot showing mean values, and bars represent SD. (a) CoQ_10_ levels in healthy controls (HC) and multiple sclerosis patients (MS), *P* = 0.001; (b) CoQ_10_ levels in HC, primary progressive (PP), secondary progressive (SP), relapsing-remitting (RR), and benign (BB) MS, *P* < 0.05; (c) CoQ_10_ in HC and MS stratified for age (49 years: median age), *P = 0.005; (d) anti-oxLDL levels in HC and MS, *P* = 0.038; (e) anti-oxLDL levels in HC, PP, SP, RR, and BB, *P* = 0.013; (f) anti-oxLDL levels in HC and MS stratified for age, *P* = 0.024.

CoQ_10_, a potent antioxidant involved in energy metabolism, was lower in MS patients than in HC subjects (*P* = 0.001) (Figure 1(a)).

BB patients had higher value of CoQ_10_ than all other groups. Post hoc analysis showed a significant difference between BB and RR (*P* < 0.05) (Figure 1(b)). Anti-oxLDL, natural antibodies reacting with bioproducts of lipid peroxidation, were higher in MS patients than in HC (*P* = 0.038) (Figure 1(d)). Post hoc analysis revealed a significant difference between HC and BB patients (*P* = 0.013) (Figure 1(e)). No statistically significant differences between patients and controls were found for GSTot, GSSG, GSH, MDA, and ROS, and there was only a trend toward decrease of serum-antioxidant power (PAO) in MS patients as compared to HC (*P* = 0.055).

In MS patients, we found significant correlations between levels of PAO and CoQ_10_ (*r* = 0.36, *P* = 0.01), PAO and GStot (*r* = 0.43, *P* < 0.01), and PAO and GSH (*r* = 0.44, *P* < 0.01). This finding confirms that CoQ_10_ and Glutathione are crucial in determining antioxidant response. Moreover, there was a negative correlation between MDA and Anti-oxLDL levels (*r* = −0.29, *P* = 0.04), suggesting these autoantibodies as contributory factor in scavenging oxidized-lipids.

It has been reported that oxidative stress naturally increases with aging, and it is known that antioxidant factors are modulated by gender [32–34]. Accordingly, we performed additional analyses after stratification for age and gender. CoQ_10_ was lower in MS patients younger than 49 years than in age-matched HC (*P* = 0.005) (Table 4 and Figure 1(c)). Conversely, anti-oxLDL level was higher in MS patients younger than 49 years than in age-matched HC (*P* = 0.024) (Table 4 and Figure 1(f)). No significant differences were found for PAO levels and other oxidative biomarkers (data not shown).

**Figure 2:**
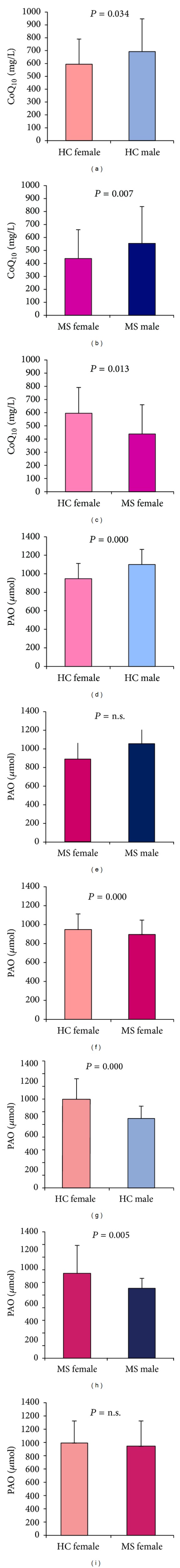
CoQ_10_, PAO, ROS levels related to gender. Bar plot showing mean values, bars represent SD. (a) CoQ_10_ levels in in Female and Male HC, *P* = 0.034; (b) CoQ_10_ levels in Female and Male MS, *P* = 0.007; (c) CoQ_10_ levels in Female HC and Female MS, *P* = 0.013; (d) PAO levels in Female and Male HC, *P* = 0.000; (e) PAO levels in Female and Male MS, *P* = n.s.; (f) CoQ_10_ levels in Female HC and Female MS, *P* = 0.000; (g) ROS levels in Female and Male HC, *P* = 0.000; (h) ROS levels in Female and Male MS, *P* = 0.005; (i) ROS levels in Female HC and Female MS, *P* = n.s.

Interestingly, we found lower antioxidant molecules (CoQ_10_, *P* = 0.007; PAO, *P* < 0.001) and higher bioproducts of oxidative stress (ROS, *P* < 0.001) in healthy females than in male counterparts (Figure 2(a)). Similar gender-related differences were found in MS patients for CoQ_10_, PAO (MS females lower than MS males, *P* = 0.034 and *P* < 0.001, resp.), and ROS (MS females higher than MS males *P* = 0.005) (Figure 2(b)). Comparing HC and patients, CoQ_10_ was lower in MS females than in HC females (*P* = 0.013) (Figure 2(c)).

Guided by the hypothesis that higher oxidative stress damage contributes to higher disease severity [9], we investigated a possible correlation between MS severity and antioxidant levels. We used EDDS scale as rough index of MS severity and we measured a correlation with any of the markers investigated. No statistical difference was found both using parametric (Pearson) and nonparametric tests (Spearman) (see supplementary Tables 7 and 8 in supplementary material available online at http://dx.doi.org/10.1155/2014/961863).

In addition, we measured disease progression rate (defined as the ratio between EDSS score and years of disease duration) and grouped patients according to the median of this value (0.36).

**Figure 3:**
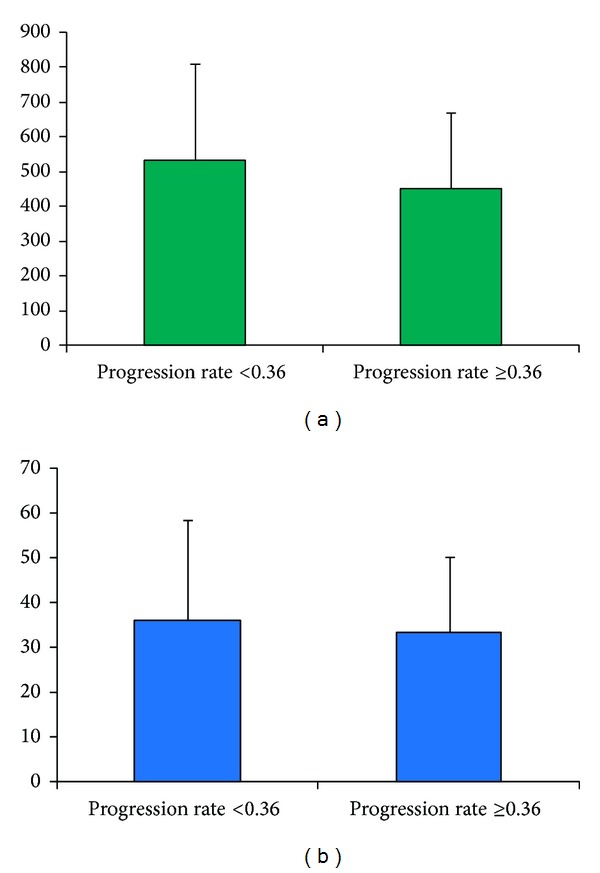
Bar plot show mean values, and bars represent SD. (a) CoQ_10_ levels in MS patients with progression rate <0.36 and progression rate >0.36 (*P* = 0.164); (b) anti-oxLDL levels in MS patients with progression rate <0.36 and progression rate >0.36 (*P* = 0.539).

We investigated a possible correlation between progression rate and CoQ_10_ or anti-oxLDL (previously reported as different between studied groups). CoQ_10_ levels showed a trend toward increase in patients with progression rate <0.36 (CoQ_10_ = 530.67 ± 281.54) than in patients with progression rate ≥0.36 (CoQ_10_ = 452.51 ± 218.54) but it was not significant (*P* = 0.164). Concerning anti-oxLDL, no statistically significant difference (*P* = 0.539) between patients with progression rate <0.36 (Anti-oxLDL 36.15 ± 22.43) and patients with progression rate ≥0.36 (Anti-oxLDL 33.45 ± 16.76) was found (data reported in Figure 3).

CoQ_10_ levels showed just a trend toward increase in patients with progression rate <0.36 (CoQ_10_ = 530.67 ± 281.54) than in patients with progression rate ≥0.36 (CoQ_10_ = 452.51 ± 218.54, *P* : n.s.). However, we found no statistically significant difference in anti-oxLDL between patients with progression rate <0.36 (anti-oxLDL 36.15 ± 22.43) and patients with progression rate ≥0.36 (anti-oxLDL 33.45 ± 16.76).

Because some interferons can modulate cellular antioxidant responses [35], we assessed a possible influence of disease-modifying drugs (i.e., IFNb and Glatimer Acetate) on oxidative stress parameters, but we found no differences in oxidative stress biomarker levels between patients treated with immunomodulant (*n* = 27) or immunosuppressive drugs (natalizumab and azathioprine, *n* = 7) or under no treatment (Table 4).

## 4. Discussion

Inflammation and neurodegeneration are intertwined processes present since the early stages of MS [3]. Among the several factors that have been involved in these mechanisms, oxidative stress damage has been the focus of numerous studies [10, 22, 23, 28, 36–39]. However, the timing, the degree, and the mechanisms by which oxidative stress contributes to MS tissue damage are still unclear. Histopathological data of Haider and colleagues [39] show that oxidative damage is massively present inside active lesions in areas of initial demyelination, at a stage regarded as a prephagocytic [40]. The same group described a deregulation of mitochondrial genes involved in redox homeostasis, which was more evident in initial lesions than in established demyelinated lesions, suggesting that oxidative stress damage associated with early mitochondria dysfunction occurs during the first stages of the disease [9]. At the beginning of MS, cellular antioxidant defenses may control oxidative stress. However, this homeostasis is lost as oxidation processes increase, typically during systemic inflammation or if the antioxidant buffer system is depleted (i.e., due to energetic failure) and tissue damage ensues. The severity of the imbalance between oxidative agents and antioxidant defenses may thus contribute to determine disease severity.

In fact, our results show that a higher antioxidant factor (CoQ_10_) is associated with a less disabling course of MS (i.e., a benign phenotype). This finding cannot be simply ascribed to a different demographic profile of benign MS (Table 2). The treatment regimen, which is clearly milder in benign MS patients, does not have any influence on CoQ_10_ or anti-oxLDL levels, because there was no difference between treated and untreated MS patients (Table 4). This finding is bolstered by the fact that the higher female/male ratio in benign MS (compared to other subgroups) should have, if anything, lowered CoQ_10_ because females have lower CoQ_10_ levels both in health controls and in MS.

CoQ_10_ is a constituent of the proton\electron transport chain, crucially involved in energy production [41]. Moreover, it acts as a primary scavenger of free radicals, protecting membrane phospholipids, proteins, and mtDNA from oxidative damage and favoring the regeneration of other antioxidants, such as tocopherol and ascorbate. It was also documented that CoQ_10_ is a calcium stabilizer, capable of alleviating calcium overload [42], and it has anti-inflammatory properties because it inhibits metalloproteinases and IL-6 production [43]. Recently, CoQ_10_ supplementation in 45 RR MS resulted in an increase of superoxide dismutase activity and a decrease in MDA levels compared with controls over 12 weeks of a randomized, double-blinded, placebo-controlled trial [44].

The higher levels of CoQ_10_ detected by our study in benign MS are consistent with a greater antioxidant buffering ability in these patients. Interestingly, CoQ_10_ difference between HC and MS patients has been seen in subjects younger than 49 years whereas it disappears in older patients. Of note, the first group shows also a shorter disease duration (*P* = 0.005) compared to the latter. This data supports the hypothesis that oxidative stress is crucial since the early stages of MS as the previously collected histopathological findings have shown [9, 11]. The different ability of patients to cope with oxidative stress may be fundamental in determining their long-term disease course.

The finding that CoQ_10_ levels are lower in females than in males, irrespective of disease status, may suggest that lower antioxidant protection can be a contributing factor in the well-known female prevalence of MS [45].

The low levels of CoQ_10_ we measured are only apparently inconsistent with the results of de Bustos et al. [46], who found no difference between MS and HC subjects. The MS population studied by these authors corresponds exactly to the one that we excluded from our study (patients during MS exacerbation). The normal CoQ_10_ levels they found in patients compared to HC could be explained by an attempt by the organism to increase antioxidant mechanisms during an inflammatory phase of disease. This phase-dependent increase might thus compensate constitutive low level of CoQ_10_.

Benign MS is associated with higher titers of anti-oxLDL antibodies. Antibodies directed against oxLDL react with bioproducts of lipid peroxidation, such as oxidized lipoproteins. These antibodies are thought to have a protective effect in atherosclerosis, where oxidation of lipoproteins is a critical event in the progression of atherogenesis [47–49].

OxLDL colocalizes with proinflammatory cells in atherosclerotic lesions and possesses a wide spectrum of highly immunogenic oxidation-specific epitopes (both lipid and protein components of LDL) [50]. Recent studies show that oxidized phospholipids favor monocyte binding to endothelial cells, thus promoting diapedesis from the blood stream [51]. Anti-oxLDL reacting with oxidized phospholipids could counteract this pathogenetic mechanism.

In addition, OxLDL is known to affect the integrity of the blood-brain barrier [52–54] and may contribute to the formation of perivascular infiltrates. Thus, increased levels of anti-OxLDL may also impair cells extravasation and inhibit the mechanisms leading to the infiltration of inflammatory cells into the brain.

Considering the high polyreactivity of anti-oxLDL antibodies [55], it is reasonable to think that they may also bind lipid epitopes on myelin debris. Benign patients may therefore have an increased ability to remove myelin remnants. Myelin debris scavenging activity could reduce an important chemoattractant signal for inflammatory cells and facilitate remyelination processes at the same time. Unfortunately, the lack of detailed data concerning these antibodies (i.e., class, immune-phenotyping of secreting B cells) makes these hypotheses only speculative at the moment. Here, we can only report the association between better prognosis and higher levels of anti-oxLDL antibodies. We cannot clarify whether these autoantibodies are an epiphenomenon of demyelination (but in this case, they should be increased in more severe cases) or are part of a healing attempt facilitating remyelination.

The higher anti-oxLDL antibodies levels that we found in benign MS are in line with the higher CoQ_10_ levels that we documented in this subgroup, suggesting that, in general, a greater antioxidant ability is associated with a milder MS course. Curiously, neither MS severity (measured by EDDS) nor disease progression (measured by progression rate) impacts these oxidative markers. This finding could favor the hypothesis that a depletion in antioxidant is not merely a consequence of mobility constraint (we should have found lower CoQ_10_ and another antioxidant associated with higher EDSS). In fact, this finding sounds to suggest antioxidant depletion as an intrinsic, and probably causative, factor associated with severe MS courses rather than its consequence.

No statistical correlation was found between anti-OxLDL and CoQ_10_ levels. This finding confirms that these variables are independent, suggesting that the mechanisms counteracting oxidative damage are diverse. Conversely, lower values of anti-OxLDL were fairly well associated with higher MDA, a marker of lipid peroxidation, suggesting the involvement of these autoantibodies in the clearance of oxidized lipids.

Another interesting finding to be highlighted is a trend toward a relative PAO deficiency in MS patients as compared to healthy controls (*P* = 0.055). Decreased plasma antioxidant capacity has been reported by several authors [10, 26, 39, 56], confirming the hypothesis that the antioxidant system is defective in MS.

MS is a CNS disorder and it is still a matter of debate how the immunological, biochemical, and oxidative abnormalities occurring in the periphery reflect the “central” phenomenon. To address this issue, we tried to investigate the same panel of oxidative biomarkers in a subgroup of patients undergoing a diagnostic spinal tap. Unfortunately, given the small amount of CSF that we considered ethical to utilize for this research, we could not obtain consistent results (data available for further analyses). Arguably, CoQ_10_ levels were below the detectability threshold even with the sophisticated method employed.

Notwithstanding these technical restrictions, we believe that our findings should trigger prospective studies aimed at addressing the potential role of an oxidative panel as a biomarker of disease course.

## 5. Conclusions

Oxidative stress markers can be different across MS courses. Indeed, benign MS patients show higher antioxidant factors, including CoQ_10_ and anti-OxLDL autoantibodies, which may confer protection against oxidative stress-driven mechanisms of neurodegeneration.

Several studies suggest that antioxidant enzymes activity is associated with the presence of neuroinflammation and oxidative damage [57]. Enhanced antioxidant mechanisms may represent a natural compensatory mechanism protecting against direct oxidative damage and avoiding an indirect inflammatory enhancing system. Higher CoQ_10_ and anti-oxLDL antibodies found in benign course MS can disclose an alternative mechanism explaining the better prognosis of this phenotype. These preliminary data should prompt additional and possibly longitudinal studies to investigate the potential role of these molecules as biomarkers and predictors of disease course.

## Supplementary Material

Correlation between oxidative stress markers and EDSS has been investigated with parametric (Pearson Coefficient) and non parametric (Correlation Coefficient ) statistical tests. No statistical correlation was found between EDSS and any biological variable as reported in Supplemt Table 7 and Table 8.

## Figures and Tables

**Table 1:** Demographic data of the studied population.

	HC	MS patients	
*N*	77	87	
F/M	42/35	52/35	*χ* = 0.5
Mean age (range)	46.6 (29–69)	44.1 (20–66)	*P* = 0.11
Median EDSS (range)	—	3.0 (0–8.0)	

HC: healthy controls; MS: multiple sclerosis patients.

**Table 2:** Demographic and clinical data of MS population.

	PP	SP	RR	BB	
*N*	20	22	32	13	
F/M	7/13	13/9	22/10	10/3	*χ* = 0.05
Mean age	45.7	45.4	40.7	47.2	*P* = 0.13
Mean EDSS	4.7	5.3	1.3	1.2	*P* < 0.001
No therapy	17	15	12	9	χ < 0.001
IFNB/GA	1	3	19	4	
Other drugs	2	4	1	0	

PP: primary progressive; SP: secondary progressive; RR: relapsing-remitting; BB: benign; HC. Other drugs: azathioprine, natalizumab, and low dose naltrexone.

**Table 3:** Oxidative stress markers' distributions in MS patients and HC.

	CoQ_10_ *μ*g/L	MDA *μ*g/L	GSTot mg/L	GSH mg/L	GSSG mg/L	PAO *μ*mol	Anti-oxLDL U/mL	ROS U Carr
MS	483.79 ± 253.75	7.58 ± 3.22	371.05 ± 120.97	317.26 ± 124.76	56.74 ± 27.63	965.03 ± 166.75	33.95 ± 19.48	315.15 ± 93.58
PP	493.25 ± 242.35	7.72 ± 3.85	363.73 ± 84.09	314.96 ± 95.63	48.22 ± 21.71	996.26 ± 142.00	31.16 ± 14.66	301.44 ± 68.30
SP	464.76 ± 182.25	7.71 ± 3.14	341.43 ± 151.54	286.84 ± 150.6	56.48 ± 27.43	931.95 ± 182.43	33.57 ± 16.46	303.191 ± 81.8
RR	429.20 ± 199.29	7.81 ± 3.05	396.28 ± 116.91	336.06 ± 109.51	62.97 ± 30.02	955.09 ± 162.09	31.89 ± 19.8	320.43 ± 121.25
BB	635.78 ± 417.05	6.60 ± 2.92	370.3 ± 121.60	326.04 ± 153.84	55.87 ± 29.75	999.85 ± 188.31	43.98 ± 27.55	341.23 ± 66.95
HC	616.13 ± 234.19	7.10 ± 2.46	360.43 ± 99.65	309.21 ± 102.28	51.21 ± 33.29	1018.25 ± 180.38	28.08 ± 15.67	315.31 ± 77.4
*P* ^†^	<**0.001**	0.30	0.55	0.66	0.26	0.06	**0.04**	0.99
*P* ^‡^	<**0.001**	0.59	0.46	0.62	0.39	0.22	0.013	0.71

Data are expressed as mean ± SD. MS: multiple sclerosis; PP: primary progressive; SP: secondary progressive; RR: relapsing-remitting; BB: benign; HC: healthy controls. *P* ^†^: comparing all MS patients with HC (Student's *t*-test was used); *P* ^‡^: each disease course was compared with HC: only BB were significantly different from HC, *P* = 0.013 (ANOVA was used).

CoQ_10_: a potent antioxidant involved in energy metabolism was lower in MS patients than in HC subjects (*P* = 0.001) (Figure 1(a)).

**Table 4:** Oxidative stress markers' distributions in MS patients according to treatment.

MS	No therapy (*n* = 53)	IFNB/GA (*n* = 27)	Other therapies (*n* = 7)	HC (*n* = 77)	*P* ^a^	*P* ^b^	*P* ^t^
Q10	536.04 ± 286.19	398.9 ± 189.48	452.35 ± 91.1	616.13 ± 234.19	0.072	0.103	0.001
MDA	7.47 ± 3.57	7.87 ± 2.92	7.58 ± 1.85	07.10 ± 02.46	0.879	0.460	0.30
Glu tot	351.37 ± 109.63	386.38 ± 127.65	453.81 ± 149.17	360.43 ± 99.65	0.063	0.113	0.55
GSH	299.93 ± 119.14	322.22 ± 120.88	413.09 ± 151.01	309.21 ± 102.28	0.056	0.102	0.66
GSSG	54.98 ± 26.59	64.16 ± 28.74	47.28 ± 24.81	51.21 ± 33.29	0.247	0.213	0.26
PAO	961.8 ± 174.98	982.73 ± 149.55	960.0 ± 168.77	1018.25 ± 180.38	0.865	0.929	0.06
Anti-oxLDL	32.93 ± 19.08	37.22 ± 19.77	32.00 ± 22.92	28.08 ± 15.67	0.631	0.517	0.04
ROS	313.85 ± 102.91	317.12 ± 76.64	320.87 ± 94.09	315.31 ± 77.4	*0.977 *	*0.940 *	0.99

No therapy: patients not under DMDs. IFNB/GA: patients using IFNBeta or glatiramer acetate. Other therapies: patients under other treatments (4 on natalizumab, 2 on azathioprine, and 1 on low dose naltrexone) HC: healthy controls. Data are expressed as mean ± SD. Using both parametric (*P* ^a^ = ANOVA) and nonparametric (*P* ^b^) tests, no statistical significant differences were found according to treatment. *P* ^t^ shows statistically significant differences between all MS patients and HC.
