# Supplementary material for: Oxidative Stress Is Differentially Present in Multiple Sclerosis Courses, Early Evident, and Unrelated to Treatment
Source: J Immunol Res. 2014 Mar 26;2014:961863. doi: 10.1155/2014/961863 (PMC3984797; doi:10.1155/2014/961863)
Supplement: Supplementary file 1 — Correlation between oxidative stress markers and EDSS has been investigated with parametric (Pearson Coefficient) and non parametric (Correlation Coefficient ) statistical tests. No statistical correlation was found between EDSS and any biological variable as reported in Supplemt Table 7 and Table 8. [file 961863.f1.pptx]

## Slide 1
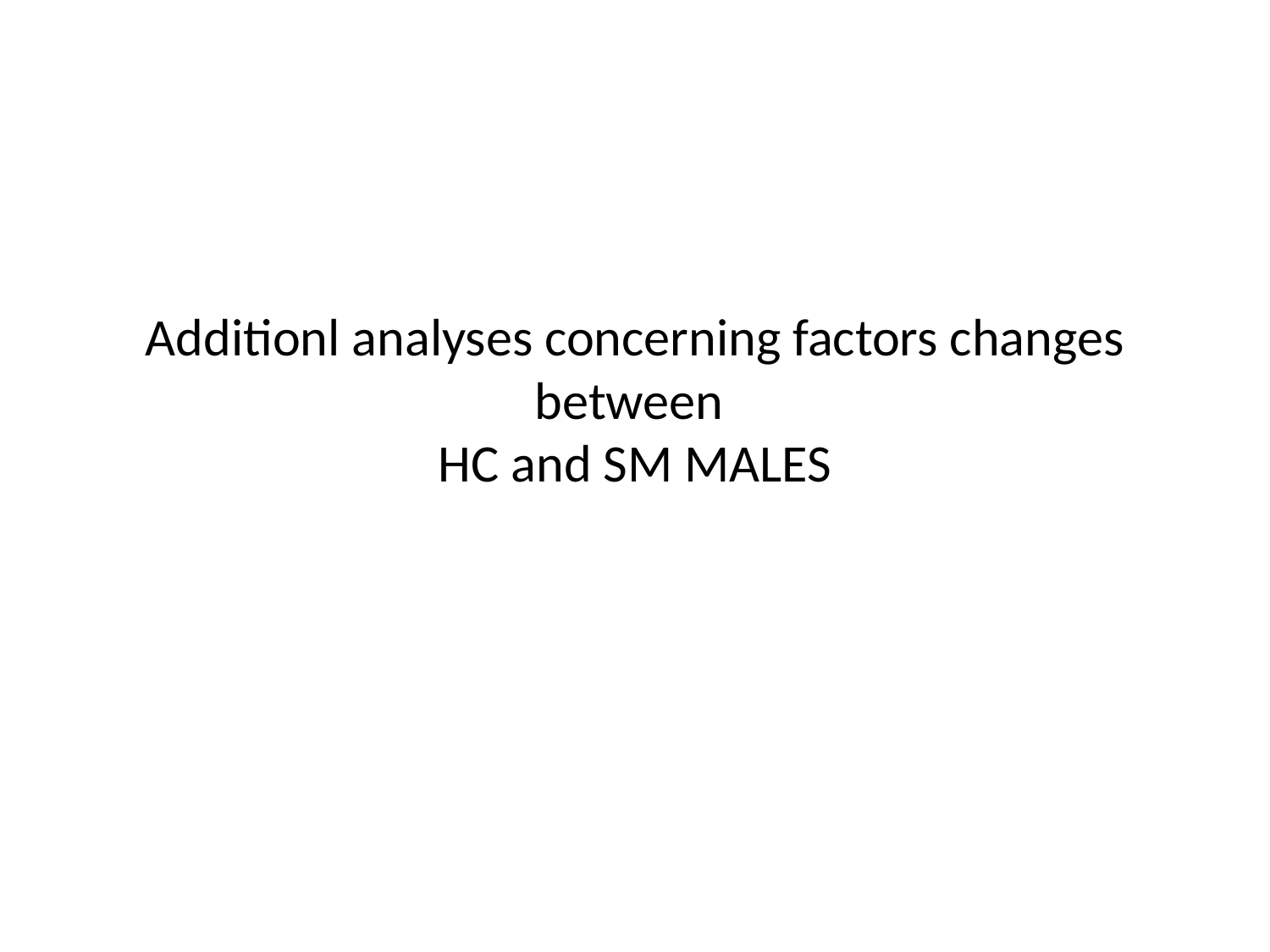

# Additionl analyses concerning factors changes between HC and SM MALES

## Slide 2
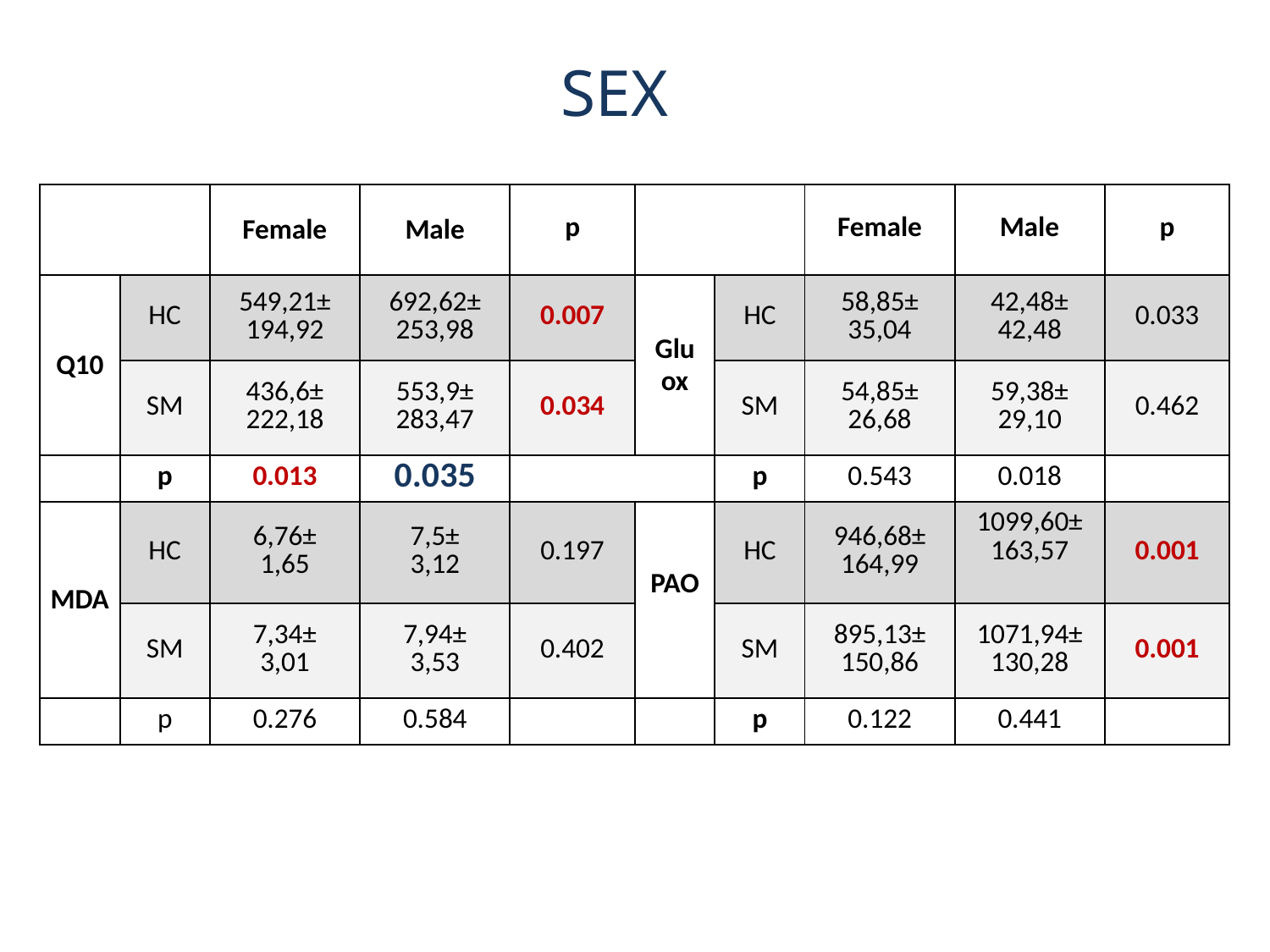

SEX
| | | Female | Male | p | | | Female | Male | p |
| --- | --- | --- | --- | --- | --- | --- | --- | --- | --- |
| Q10 | HC | 549,21± 194,92 | 692,62± 253,98 | 0.007 | Glu ox | HC | 58,85± 35,04 | 42,48± 42,48 | 0.033 |
| | SM | 436,6± 222,18 | 553,9± 283,47 | 0.034 | | SM | 54,85± 26,68 | 59,38± 29,10 | 0.462 |
| | p | 0.013 | 0.035 | | | p | 0.543 | 0.018 | |
| MDA | HC | 6,76± 1,65 | 7,5± 3,12 | 0.197 | PAO | HC | 946,68± 164,99 | 1099,60± 163,57 | 0.001 |
| | SM | 7,34± 3,01 | 7,94± 3,53 | 0.402 | | SM | 895,13± 150,86 | 1071,94± 130,28 | 0.001 |
| | p | 0.276 | 0.584 | | | p | 0.122 | 0.441 | |

## Slide 3
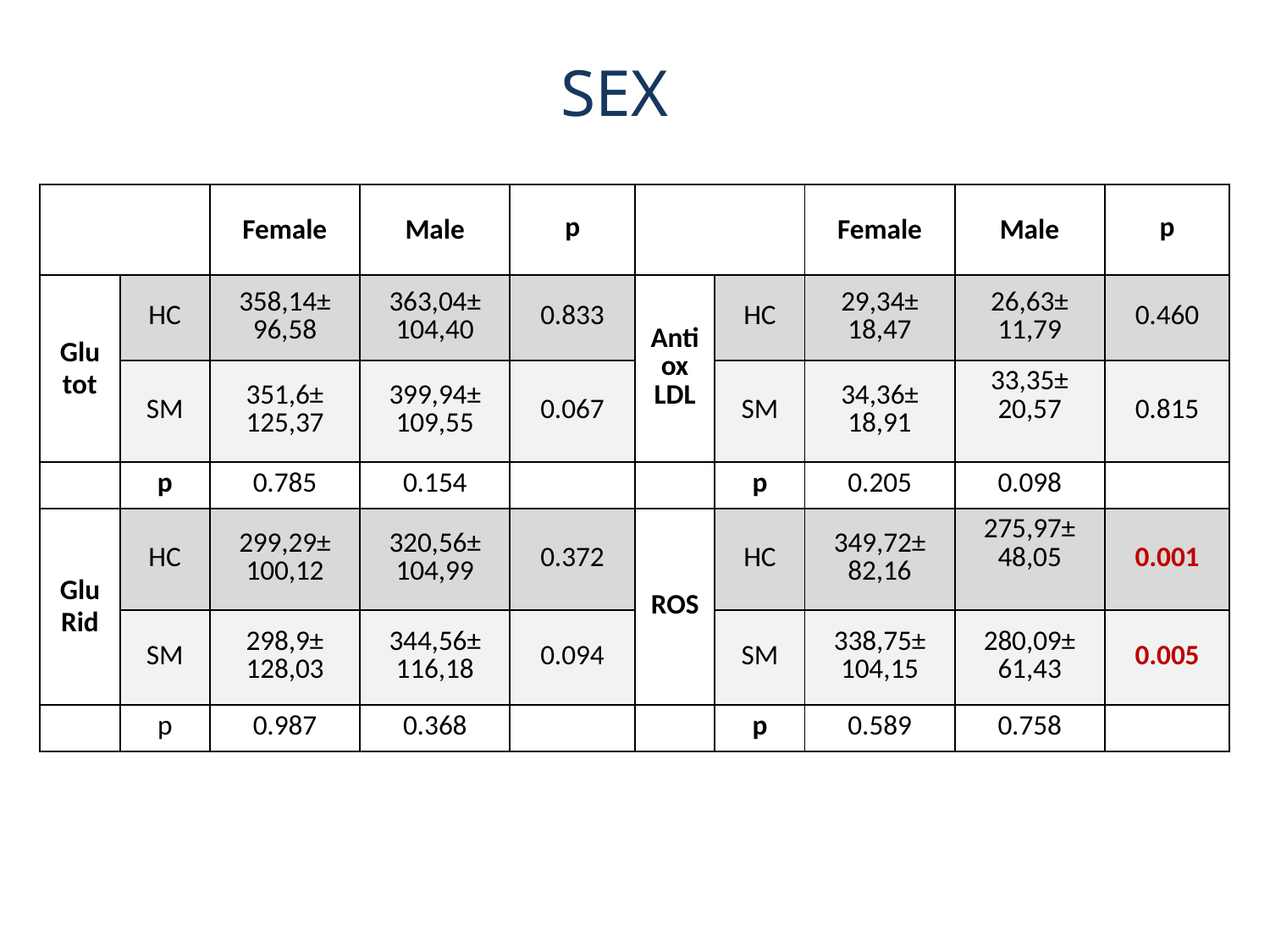

SEX
| | | Female | Male | p | | | Female | Male | p |
| --- | --- | --- | --- | --- | --- | --- | --- | --- | --- |
| Glu tot | HC | 358,14± 96,58 | 363,04± 104,40 | 0.833 | Antiox LDL | HC | 29,34± 18,47 | 26,63± 11,79 | 0.460 |
| | SM | 351,6± 125,37 | 399,94± 109,55 | 0.067 | | SM | 34,36± 18,91 | 33,35± 20,57 | 0.815 |
| | p | 0.785 | 0.154 | | | p | 0.205 | 0.098 | |
| Glu Rid | HC | 299,29± 100,12 | 320,56± 104,99 | 0.372 | ROS | HC | 349,72± 82,16 | 275,97± 48,05 | 0.001 |
| | SM | 298,9± 128,03 | 344,56± 116,18 | 0.094 | | SM | 338,75± 104,15 | 280,09± 61,43 | 0.005 |
| | p | 0.987 | 0.368 | | | p | 0.589 | 0.758 | |
